# Crystal structure and Hirshfeld surface analysis of (*E*)-4-{[2,2-di­chloro-1-(4-meth­oxy­phen­yl)ethen­yl]diazen­yl}benzo­nitrile

**DOI:** 10.1107/S2056989019009642

**Published:** 2019-07-16

**Authors:** Mehmet Akkurt, Namiq Q. Shikhaliyev, Ulviyya F. Askerova, Sevinc H. Mukhtarova, Gunay Z. Mammadova, Flavien A. A. Toze

**Affiliations:** a Department of Physics, Faculty of Sciences, Erciyes University, 38039 Kayseri, Turkey; b Organic Chemistry Department, Baku State University, Z. Xalilov str. 23, Az, 1148 Baku, Azerbaijan; c Department of Chemistry, Faculty of Sciences, University of Douala, PO Box 24157, Douala, Republic of Cameroon

**Keywords:** crystal structure, 4-meth­oxy­phenyl ring, benzo­nitrile, face-to-face π–π stacking inter­actions, Hirshfeld surface analysis

## Abstract

In the title compound, C_16_H_11_Cl_2_N_3_O, the 4-meth­oxy-substituted benzene ring makes a dihedral angle of 41.86 (9)° with the benzene ring of the benzo­nitrile group. In the crystal, mol­ecules are linked into layers parallel to (020) by C—H⋯O contacts and face-to-face π–π stacking inter­actions [centroid–centroid distances = 3.9116 (14) and 3.9118 (14) Å] between symmetry-related aromatic rings along the *a-*axis direction. A Hirshfeld surface analysis indicates that the most important contributions to the crystal packing are from Cl⋯H/H⋯Cl (22.8%), H⋯H (21.4%), N⋯H/H⋯N (16.1%), C⋯H/H⋯C (14.7%) and C⋯C (9.1%) inter­actions.

## Abstract 2

In the crystal structure, the mol­ecules are linked into layers parallel to the (020) plane by C—H⋯O contacts and face-to-face π–π stacking inter­actions between symmetry-related aromatic rings along the *a-*axis direction.

## Chemical context   

Weak inter­actions, such as hydrogen, aerogen, halogen, chalcogen, pnicogen, tetrel and icosa­gen bonds, as well as *n*–π*, *π–π* stacking, *π*–cation, *π*–anion and hydro­phobic inter­actions, can control or organize the conformation, aggregation, tertiary and quaternary structure of the mol­ecule, its reactivity, stabilization and other properties (Asadov *et al.*, 2016 ▸; Maharramov *et al.*, 2010 ▸; Mahmudov *et al.*, 2013 ▸, 2014*a* ▸,*b* ▸, 2015 ▸, 2017*a* ▸,*b* ▸, 2019 ▸; Shixaliyev *et al.*, 2013 ▸, 2014 ▸). The functionalization of azo/hydrazone ligands with non-covalent bond-donor or acceptor sites greatly affects their coordination ability and the catalytic activity of the corresponding coordination compounds (Akbari *et al.*, 2017 ▸; Gurbanov *et al.*, 2018 ▸; Karmakar *et al.*, 2016 ▸; Kopylovich *et al.*, 2011*a* ▸,*b* ▸; Ma *et al.*, 2017*a* ▸,*b* ▸; Mahmoudi *et al.*, 2016 ▸, 2017*a* ▸,*b* ▸,*c* ▸, 2018*a* ▸,*b* ▸,*c* ▸). In our previous work, we have attached chloro atoms to dye mol­ecules, which lead to halogen bonding (Atioğlu *et al.*, 2019 ▸; Maharramov *et al.*, 2018 ▸; Shixaliyev *et al.*, 2018 ▸, 2019 ▸). In a continuation of this work, we have functionalized a new azo dye, (*E*)-4-{[2,2-di­chloro-1-(4-meth­oxy­phen­yl)ethen­yl]diazen­yl}benzo­nitrile, which provides weak C—H⋯O inter­molecular hydrogen bonds.
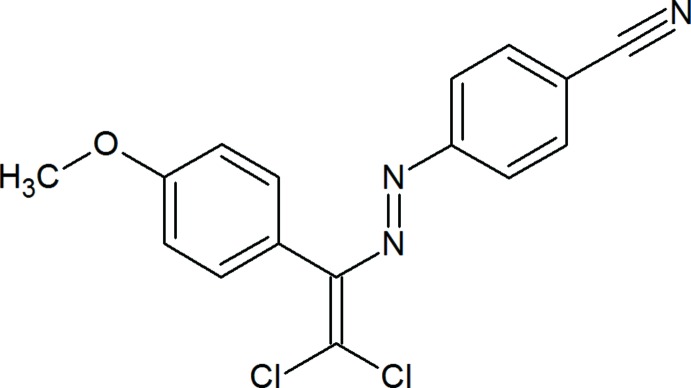


## Structural commentary   

**Figure 1:**
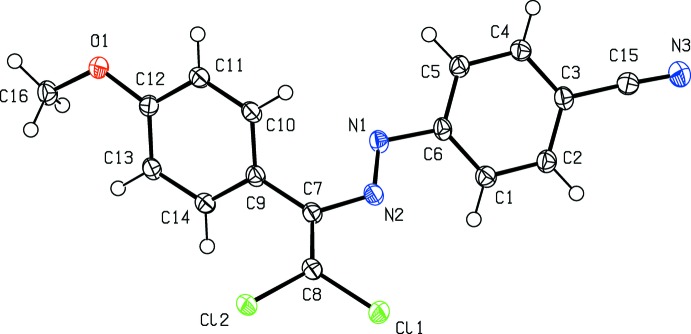
The mol­ecular structure of the title compound, with the atom labelling. Displacement ellipsoids are drawn at the 50% probability level.

In the title compound, (Fig. 1 ▸), the dihedral angle between the 4-meth­oxy-substituted benzene ring and the benzene ring of the benzo­nitrile moiety is 41.86 (9)°. The C1—C6—N1—N2, C6—N1—N2—C7, N1—N2—C7—C8, N2—C7—C8—Cl1, N2—C7—C8—Cl2, Cl1—C8—C7—C9 and C8—C7—C9—C14 torsion angles of 24.8 (2), −178.37 (15), −176.77 (17), −2.2 (2), 178.27 (14), −176.26 (14) and −52.1 (3)°, respectively, describe the essentially planar conformation of the di­chloro-vinyl­diazenyl moiety. Bond lengths and angles are within normal ranges and are comparable to those observed in related structures such as (*E*)-1-[2,2-di­chloro-1-(4-nitro­phen­yl)ethen­yl]-2-(4-fluoro­phen­yl)diazene (Atioğlu *et al.*, 2019 ▸), (2*E*)-1-(2-hy­droxy-5-methyl­phen­yl)-3-(4-meth­oxy­phen­yl)prop-2-en-1-one (Fun *et al.*, 2011*a* ▸), (2*E*)-3-(3-benzyl­oxyphen­yl)-1-(2-hy­droxy-5-methyl­phen­yl)prop-2-en-1-one (Fun *et al.*, 2011*b* ▸), (2*E*)-3-[3-(benz­yloxy)phen­yl]-1-(2-hy­droxy­phen­yl)prop-2-en-1-one (Fun *et al.*, 2011*c* ▸), (2*E*)-1-(2,5-di­meth­oxy­phen­yl)-3-(3-nitro­phen­yl)prop-2-en-1-one (Fun *et al.*, 2011*d* ▸) and (2*E*)-3-(3-nitro­phen­yl)-1-[4-(piperidin-1-yl)phen­yl]prop-2-en-1-one (Fun *et al.*, 2012 ▸).

## Supra­molecular features and Hirshfeld surface analysis   

**Figure 2:**
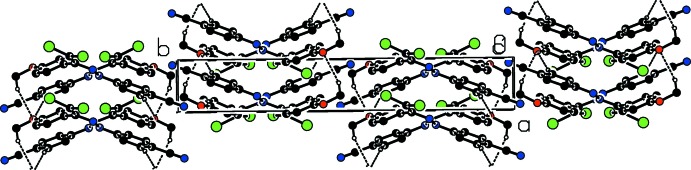
A view of the crystal packing of the title compound. The weak C—H⋯O inter­actions are shown as dashed lines and H atoms not involved in hydrogen bonding have been omitted for clarity.

In the crystal, the mol­ecules are linked into layers parallel to the (020) plane by C—H⋯O contacts and face-to-face π–π stacking inter­actions [centroid-centroid distances = 3.9116 (14) and 3.9118 (14) Å] along the *a*-axis between the same aromatic rings (Table 1 ▸; Figs. 2 ▸ and 3 ▸). These mol­ecular layers are held together by weak van der Waals forces.

**Figure 4:**
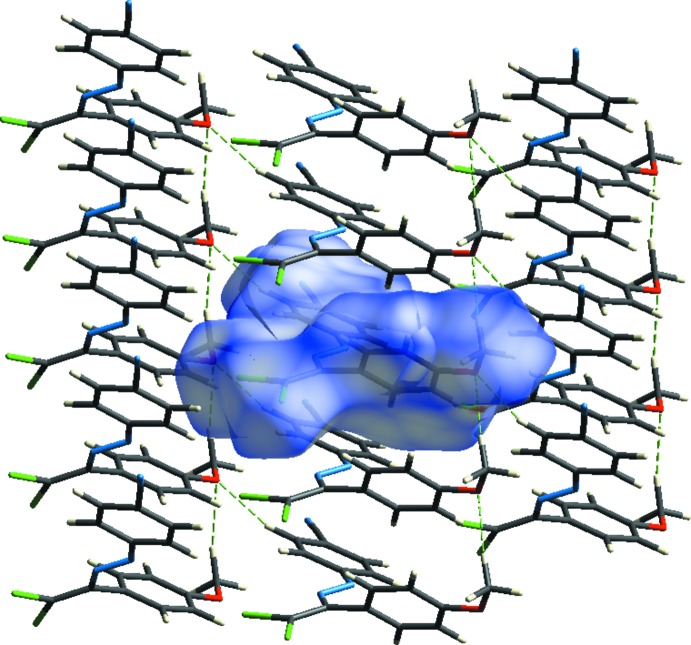
A view of the three-dimensional Hirshfeld surface of the title compound mapped over *d*_norm_ showing the C—H⋯O inter­actions (dashed lines).

**Figure 5:**
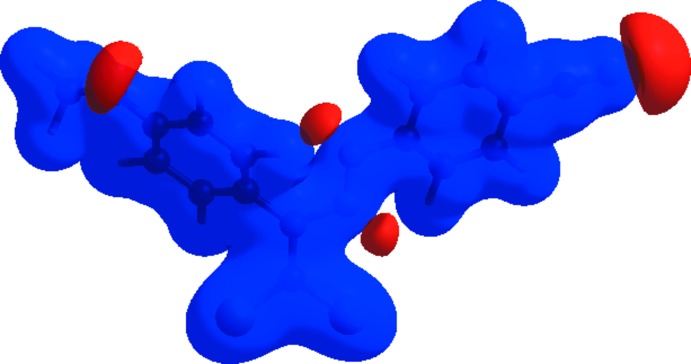
View of the three-dimensional Hirshfeld surface of the title compound plotted over electrostatic potential energy in the range −0.0500 to 0.0500 a.u. using the STO-3 G basis set at the Hartree–Fock level of theory. Hydrogen-bond donors and acceptors are shown as blue and red regions around the atoms, corresponding to positive and negative potentials, respectively.

**Figure 6:**
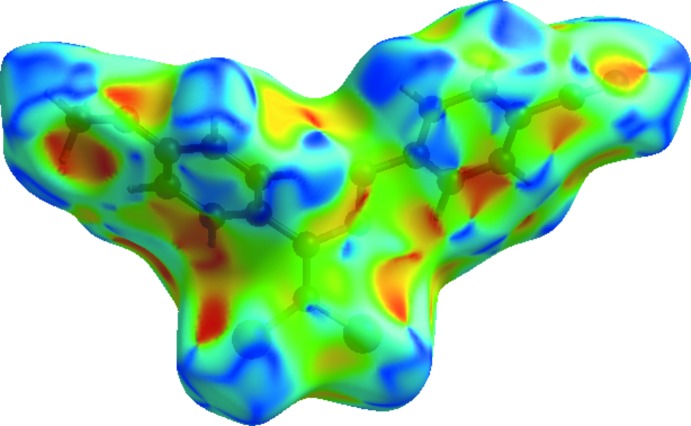
Hirshfeld surface of the title compound plotted over shape-index.

Hirshfeld surfaces and fingerprint plots were generated for the title compound using *CrystalExplorer* (McKinnon *et al.*, 2007 ▸) to qu­antify and visualize the inter­molecular inter­actions and to explain the observed crystal packing. The Hirshfeld surface mapped over *d*_norm_ using a standard surface resolution with a fixed colour scale of −0.1603 (red) to 1.2420 (blue) a.u. is shown in Fig. 4 ▸. The dark-red spots on the *d*_norm_ surface arise as a result of short inter­atomic contacts (Table 2 ▸), while the other weaker inter­molecular inter­actions appear as light-red spots. The red points, which represent closer contacts and negative *d*_norm_ values on the surface, correspond to the C—H⋯O inter­actions. The Hirshfeld surface mapped over electrostatic potential (Spackman *et al.*, 2008 ▸) is shown in Fig. 5 ▸. The red regions indicate atoms with the potential to be hydrogen-bond acceptors (negative electrostatic potential), while blue regions indicate atoms with positive electrostatic potential, *i.e.* hydrogen-bond donors. The shape-index of the Hirshfeld surface is a tool to visualize the π–π stacking by the presence of adjacent red and blue triangles; if there are no adjacent red and/or blue triangles, then there are no π–π inter­actions. Fig. 6 ▸ clearly suggest that there are π–π inter­actions in the title compound.

**Figure 7:**
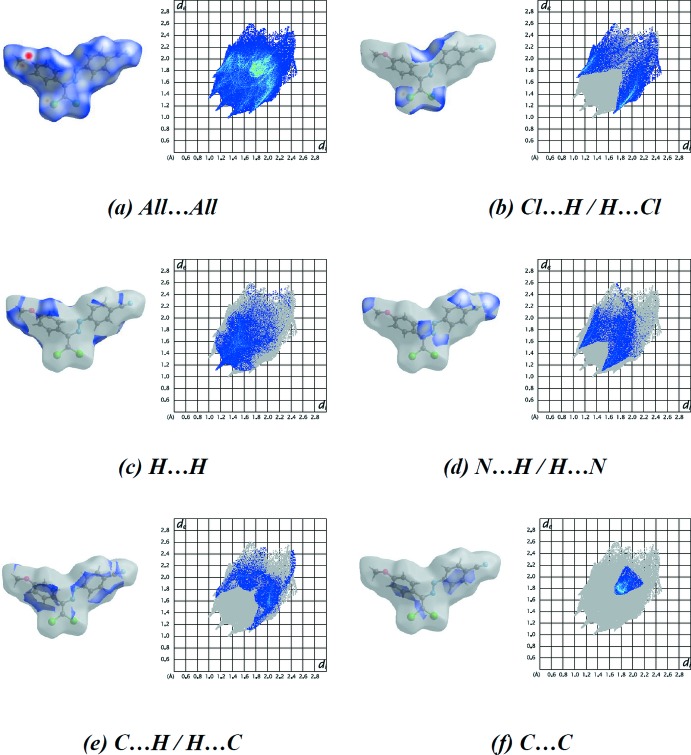
The Hirshfeld surface representations and two-dimensional fingerprint plots of the title compound showing all inter­actions, and the most significant individual types of inter­actions.

The percentage contributions of the various contacts to the total Hirshfeld surface are shown in the two dimensional fingerprint plots in Table 3 ▸. The reciprocal Cl⋯H/H⋯Cl inter­actions appear as two symmetrical broad wings with *d*_e_ + *d*_i_ ≃ 2.8 Å and contribute 22.8% to the Hirshfeld surface (Fig. 7 ▸ *b*). The H⋯H inter­actions appear in the middle of the scattered points in the two dimensional fingerprint plots, with an overall contribution to the Hirshfeld surface of 21.4% (Fig. 7 ▸ *c*). The N⋯H/H⋯N and C⋯H/H⋯C inter­actions also appear as two symmetrical broad wings with *d*_e_ + *d*_i_ ≃ 2.6 and 2.8 Å, respectively, and contribute 16.1 and 14.7%, respectively, to the Hirshfeld surface (Fig. 7 ▸ *d,e*). The C⋯C inter­actions appear in the middle of the scattered points in the two-dimensional fingerprint plots with an overall contribution to the Hirshfeld surface of 9.1% (Fig. 7 ▸ *f*). The small percentage contributions from the other different inter­atomic contacts to the Hirshfeld surfaces are listed in Table 3 ▸. The large number of Cl⋯H/H⋯Cl, H⋯H, N⋯H/H⋯N, C⋯H/H⋯C and C⋯C inter­actions suggest that van der Waals inter­actions and hydrogen bonding play the major roles in the crystal packing (Hathwar *et al.*, 2015 ▸).

## Synthesis and crystallization   

The title compound was synthesized according to the reported method (Atioğlu *et al.*, 2019 ▸; Maharramov *et al.*, 2018 ▸; Shikhaliyev *et al.*, 2018 ▸, 2019 ▸). A 20 mL screw-neck vial was charged with DMSO (10 mL), (*E*)-4-[2-(4-meth­oxy­benzyl­idene)hydrazine­yl]benzo­nitrile (251 mg, 1 mmol), tetra­methyl­ethylenedi­amine (TMEDA; 295 mg, 2.5 mmol), CuCl (2 mg, 0.02 mmol) and CCl_4_ (20 mmol, 10 equiv). After 1–3 h (after TLC analysis showed complete consumption of the corresponding Schiff base), the reaction mixture was poured into an 0.01 *M* solution of HCl (100 mL, pH = 2–3) and extracted with di­chloro­methane (3x20 mL). The combined organic phase was washed with water (3x50 mL), brine (30 mL), dried over anhydrous Na_2_SO_4_ and concentrated *in vacuo* of the rotary evaporator. The residue was purified by column chromatography on silica gel using appropriate mixtures of hexane and di­chloro­methane (3/1–1/1), giving an orange solid (63%); m.p. 471 K. Analysis calculated for C_16_H_11_Cl_2_N_3_O (*M* = 332.18): C 57.85, H 3.34, N 12.65; found: C 57.78, H 3.29, N 12.58%. ^1^H NMR (300 MHz, CDCl_3_) *δ* 3.83–3.93 (3H, OCH_3_), 6.89–7.70 (8H, Ar). ^13^C NMR (75 MHz, CDCl_3_) δ 153.89, 133.85, 133.14, 130.54, 130.37, 130.34, 115.49, 115.00, 113.80, 55.51, 29.72, 14.15. ESI–MS: *m*/*z*: 333.17 [*M* + H]^+^.

## Refinement   

Crystal data, data collection and structure refinement details are summarized in Table 4 ▸. The H atoms of aromatic and methyl groups were placed in calculated positions (C—H = 0.95 and 0.98 Å, respectively) and refined using a riding model with *U*_iso_= 1.2*U*_eq_(C-aromatic) and 1.5*U*_eq_(C-meth­yl).

## Supplementary Material

Crystal structure: contains datablock(s) I. DOI: 10.1107/S2056989019009642/lh5909sup1.cif

Structure factors: contains datablock(s) I. DOI: 10.1107/S2056989019009642/lh5909Isup2.hkl

Supporting information file. DOI: 10.1107/S2056989019009642/lh5909Isup3.cml

CCDC reference: 1938782

Additional supporting information:  crystallographic information; 3D view; checkCIF report

## Figures and Tables

**Figure 3:**
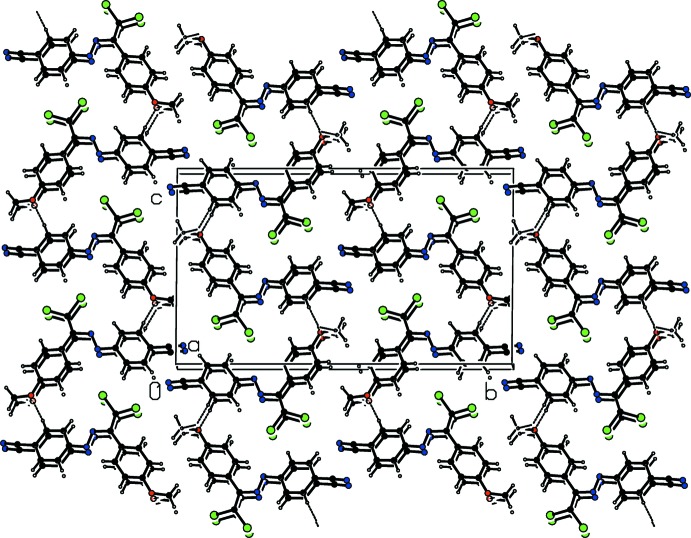
A packing diagram of the title compound, viewed along the *a* axis. The C—H⋯O inter­actions are shown as dashed lines.

**Table 1:** Hydrogen-bond geometry (Å, °)

*D*—H⋯*A*	*D*—H	H⋯*A*	*D*⋯*A*	*D*—H⋯*A*
C2—H2⋯O1^i^	0.95	2.47	3.391 (2)	165
C16—H16*C*⋯O1^ii^	0.98	2.59	3.516 (3)	158

Symmetry codes: (i) 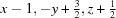; (ii) 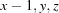.

**Table 2:** Summary of short inter­atomic contacts (Å) in the title compound

Contact	Distance	Symmetry operation
Cl1⋯H5	3.05	1 + *x*, 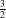 − *y*, 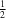 + *z*
Cl1⋯H10	2.98	*x*, 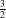 − *y*, 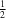 + *z*
O1⋯H16*C*	2.59	1 + *x*, *y*, *z*
Cl2⋯N3	3.462	1 − *x*, 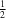 + *y*, 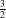 − *z*
H16*B*⋯C13	2.90	2 − *x*, 2 − *y*, 1 − *z*
O1⋯H2	2.47	1 + *x*, 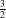 − *y*, − 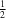 + *z*
H4⋯N3	2.78	−*x*, 1 − *y*, 1 − *z*
H4⋯N3	2.82	1 − *x*, 1 − *y*, 1 − *z*
H13⋯H13	2.52	1 − *x*, 2 − *y*, 1 − *z*

**Table 3:** Percentage contributions of inter­atomic contacts to the Hirshfeld surface

Contact	Percentage contribution
Cl⋯H/H⋯Cl	22.8
H⋯H	21.4
N⋯H/H⋯N	16.1
C⋯H/H⋯C	14.7
C⋯C	9.1
O⋯H/H⋯O	5.3
N⋯C/C⋯N	4.2
Cl⋯N/N⋯Cl	2.6
Cl⋯C/C⋯Cl	1.7
Cl⋯Cl	1.6
C⋯O/O⋯C	0.4
N⋯N	0.2

**Table 4:** Experimental details

Crystal data	
Chemical formula	C_16_H_11_Cl_2_N_3_O
*M*_r_	332.18
Crystal system, space group	Monoclinic, *P*2_1_/*c*
Temperature (K)	100
*a*, *b*, *c* (Å)	3.9117 (8), 25.109 (5), 14.968 (3)
β (°)	97.07 (3)
*V* (Å^3^)	1459.0 (5)
*Z*	4
Radiation type	Synchrotron, λ = 0.80246 Å
μ (mm^−1^)	0.63
Crystal size (mm)	0.25 × 0.05 × 0.03

Data collection	
Diffractometer	Rayonix SX165 CCD
Absorption correction	Multi-scan (*SCALA*; Evans, 2006 ▸)
*T*_min_, *T*_max_	0.850, 0.975
No. of measured, independent and observed [*I* > 2σ(*I*)] reflections	22750, 3144, 2978
*R*_int_	0.068
(sin θ/λ)_max_ (Å^−1^)	0.639

Refinement	
*R*[*F* ^2^ > 2σ(*F* ^2^)], *wR*(*F* ^2^), *S*	0.045, 0.123, 1.07
No. of reflections	3144
No. of parameters	201
H-atom treatment	H-atom parameters constrained
Δρ_max_, Δρ_min_ (e Å^−3^)	0.30, −0.46

Computer programs: *Marccd* (Doyle, 2011 ▸), *iMosflm* (Battye *et al.*, 2011 ▸), SHELXLS97 (Sheldrick, 2008 ▸), *SHELXL2016* (Sheldrick, 2015 ▸), *ORTEP-3 for Windows* (Farrugia, 2012 ▸) and *PLATON* (Spek, 2009 ▸).

## supplementary crystallographic information

### Crystal data

**Table:**

C_16_H_11_Cl_2_N_3_O	*F*(000) = 680
*M**_r_* = 332.18	*D*_x_ = 1.512 Mg m^−^^3^
Monoclinic, *P*2_1_/*c*	Synchrotron radiation, λ = 0.80246 Å
*a* = 3.9117 (8) Å	Cell parameters from 600 reflections
*b* = 25.109 (5) Å	θ = 1.8–30.0°
*c* = 14.968 (3) Å	µ = 0.63 mm^−^^1^
β = 97.07 (3)°	*T* = 100 K
*V* = 1459.0 (5) Å^3^	Needle, orange
*Z* = 4	0.25 × 0.05 × 0.03 mm

### Data collection

**Table:**

Rayonix SX165 CCD diffractometer	2978 reflections with *I* > 2σ(*I*)
/f scan	*R*_int_ = 0.068
Absorption correction: multi-scan (Scala; Evans, 2006)	θ_max_ = 30.9°, θ_min_ = 1.8°
*T*_min_ = 0.850, *T*_max_ = 0.975	*h* = −4→4
22750 measured reflections	*k* = −32→32
3144 independent reflections	*l* = −19→19

### Refinement

**Table:**

Refinement on *F*^2^	Hydrogen site location: inferred from neighbouring sites
Least-squares matrix: full	H-atom parameters constrained
*R*[*F*^2^ > 2σ(*F*^2^)] = 0.045	*w* = 1/[σ^2^(*F*_o_^2^) + (0.066*P*)^2^ + 0.9448*P*] where *P* = (*F*_o_^2^ + 2*F*_c_^2^)/3
*wR*(*F*^2^) = 0.123	(Δ/σ)_max_ = 0.001
*S* = 1.07	Δρ_max_ = 0.30 e Å^−^^3^
3144 reflections	Δρ_min_ = −0.46 e Å^−^^3^
201 parameters	Extinction correction: SHELXL2018 (Sheldrick, 2015), Fc^*^=kFc[1+0.001xFc^2^λ^3^/sin(2θ)]^-1/4^
0 restraints	Extinction coefficient: 0.049 (5)

### Special details

**Table:**

Geometry. All esds (except the esd in the dihedral angle between two l.s. planes) are estimated using the full covariance matrix. The cell esds are taken into account individually in the estimation of esds in distances, angles and torsion angles; correlations between esds in cell parameters are only used when they are defined by crystal symmetry. An approximate (isotropic) treatment of cell esds is used for estimating esds involving l.s. planes.

### Fractional atomic coordinates and isotropic or equivalent isotropic displacement parameters (Å^2^)

**Table:**

	*x*	*y*	*z*	*U*_iso_*/*U*_eq_	
C1	0.4566 (5)	0.66476 (7)	0.67302 (12)	0.0215 (4)	
H1	0.463381	0.687885	0.723440	0.026*	
C2	0.3294 (5)	0.61359 (7)	0.67752 (13)	0.0221 (4)	
H2	0.244963	0.601450	0.730780	0.026*	
C3	0.3261 (5)	0.57981 (7)	0.60312 (13)	0.0215 (4)	
C4	0.4409 (5)	0.59720 (7)	0.52351 (12)	0.0228 (4)	
H4	0.436434	0.574023	0.473208	0.027*	
C5	0.5621 (5)	0.64912 (7)	0.51906 (12)	0.0214 (4)	
H5	0.636172	0.661965	0.464913	0.026*	
C6	0.5748 (5)	0.68213 (7)	0.59378 (12)	0.0197 (4)	
C7	0.9517 (5)	0.80678 (7)	0.65882 (12)	0.0199 (4)	
C8	1.0851 (5)	0.82299 (7)	0.74254 (13)	0.0218 (4)	
C9	0.9344 (5)	0.84019 (7)	0.57700 (12)	0.0199 (4)	
C10	1.0545 (5)	0.82125 (7)	0.49838 (13)	0.0210 (4)	
H10	1.155098	0.786823	0.498109	0.025*	
C11	1.0283 (5)	0.85197 (7)	0.42152 (12)	0.0213 (4)	
H11	1.108147	0.838403	0.368576	0.026*	
C12	0.8849 (5)	0.90295 (7)	0.42105 (12)	0.0198 (4)	
C13	0.7626 (5)	0.92238 (7)	0.49813 (13)	0.0208 (4)	
H13	0.662374	0.956840	0.498230	0.025*	
C14	0.7886 (5)	0.89082 (7)	0.57518 (13)	0.0210 (4)	
H14	0.704725	0.904154	0.627752	0.025*	
C15	0.1992 (5)	0.52632 (8)	0.61044 (13)	0.0249 (4)	
C16	0.7123 (5)	0.98149 (7)	0.33590 (13)	0.0246 (4)	
H16A	0.731494	0.997143	0.276745	0.037*	
H16B	0.823240	1.005003	0.383095	0.037*	
H16C	0.468596	0.977180	0.343527	0.037*	
N1	0.7074 (4)	0.73473 (6)	0.58427 (10)	0.0207 (3)	
N2	0.8218 (4)	0.75417 (6)	0.65946 (10)	0.0212 (3)	
N3	0.1007 (5)	0.48385 (7)	0.61986 (13)	0.0345 (4)	
O1	0.8781 (4)	0.93062 (5)	0.34222 (9)	0.0233 (3)	
Cl1	1.07739 (13)	0.78390 (2)	0.83659 (3)	0.02703 (18)	
Cl2	1.27416 (13)	0.88356 (2)	0.76669 (3)	0.02496 (18)	

### Atomic displacement parameters (Å^2^)

**Table:**

	*U*^11^	*U*^22^	*U*^33^	*U*^12^	*U*^13^	*U*^23^
C1	0.0237 (9)	0.0195 (9)	0.0207 (9)	0.0005 (7)	−0.0001 (7)	0.0001 (7)
C2	0.0246 (9)	0.0192 (9)	0.0220 (9)	0.0002 (7)	0.0009 (7)	0.0028 (7)
C3	0.0234 (9)	0.0166 (8)	0.0234 (9)	−0.0005 (7)	−0.0012 (7)	0.0016 (7)
C4	0.0285 (10)	0.0197 (9)	0.0193 (9)	−0.0018 (7)	−0.0009 (7)	−0.0006 (7)
C5	0.0242 (9)	0.0195 (8)	0.0193 (8)	−0.0005 (7)	−0.0021 (7)	0.0024 (7)
C6	0.0207 (9)	0.0154 (8)	0.0218 (9)	0.0007 (7)	−0.0020 (7)	0.0027 (6)
C7	0.0223 (9)	0.0159 (8)	0.0211 (9)	0.0008 (7)	0.0016 (7)	−0.0007 (6)
C8	0.0257 (9)	0.0173 (8)	0.0223 (9)	0.0005 (7)	0.0025 (7)	−0.0010 (7)
C9	0.0215 (9)	0.0167 (8)	0.0207 (9)	−0.0021 (7)	−0.0003 (7)	−0.0006 (6)
C10	0.0244 (9)	0.0159 (8)	0.0221 (9)	0.0002 (7)	0.0000 (7)	−0.0026 (7)
C11	0.0244 (9)	0.0181 (8)	0.0211 (9)	−0.0005 (7)	0.0016 (7)	−0.0021 (7)
C12	0.0221 (9)	0.0186 (8)	0.0179 (8)	−0.0029 (7)	−0.0007 (7)	0.0012 (6)
C13	0.0237 (9)	0.0150 (8)	0.0231 (9)	−0.0004 (7)	0.0006 (7)	−0.0003 (7)
C14	0.0247 (9)	0.0178 (8)	0.0201 (9)	−0.0004 (7)	0.0017 (7)	−0.0018 (6)
C15	0.0294 (10)	0.0231 (10)	0.0217 (9)	−0.0028 (8)	0.0011 (7)	−0.0002 (7)
C16	0.0290 (10)	0.0182 (9)	0.0254 (10)	0.0013 (7)	−0.0015 (7)	0.0030 (7)
N1	0.0225 (8)	0.0161 (7)	0.0227 (8)	0.0004 (6)	−0.0004 (6)	0.0013 (6)
N2	0.0251 (8)	0.0164 (7)	0.0212 (8)	−0.0003 (6)	−0.0007 (6)	−0.0006 (6)
N3	0.0490 (12)	0.0242 (9)	0.0304 (9)	−0.0092 (8)	0.0046 (8)	−0.0008 (7)
O1	0.0307 (7)	0.0186 (6)	0.0203 (7)	0.0020 (5)	0.0018 (5)	0.0023 (5)
Cl1	0.0386 (3)	0.0223 (3)	0.0191 (3)	−0.00365 (18)	−0.00068 (19)	0.00226 (16)
Cl2	0.0343 (3)	0.0185 (3)	0.0213 (3)	−0.00454 (17)	0.00074 (18)	−0.00278 (15)

### Geometric parameters (Å, º)

**Table:**

C1—C2	1.382 (3)	C9—C14	1.392 (2)
C1—C6	1.395 (3)	C9—C10	1.403 (3)
C1—H1	0.9500	C10—C11	1.378 (3)
C2—C3	1.399 (3)	C10—H10	0.9500
C2—H2	0.9500	C11—C12	1.397 (3)
C3—C4	1.394 (3)	C11—H11	0.9500
C3—C15	1.441 (3)	C12—O1	1.367 (2)
C4—C5	1.392 (3)	C12—C13	1.391 (3)
C4—H4	0.9500	C13—C14	1.393 (3)
C5—C6	1.388 (3)	C13—H13	0.9500
C5—H5	0.9500	C14—H14	0.9500
C6—N1	1.433 (2)	C15—N3	1.149 (3)
C7—C8	1.359 (3)	C16—O1	1.430 (2)
C7—N2	1.416 (2)	C16—H16A	0.9800
C7—C9	1.479 (2)	C16—H16B	0.9800
C8—Cl2	1.7103 (19)	C16—H16C	0.9800
C8—Cl1	1.7196 (19)	N1—N2	1.257 (2)
			
C2—C1—C6	119.40 (17)	C10—C9—C7	121.08 (16)
C2—C1—H1	120.3	C11—C10—C9	120.81 (17)
C6—C1—H1	120.3	C11—C10—H10	119.6
C1—C2—C3	119.55 (18)	C9—C10—H10	119.6
C1—C2—H2	120.2	C10—C11—C12	120.36 (17)
C3—C2—H2	120.2	C10—C11—H11	119.8
C4—C3—C2	121.20 (17)	C12—C11—H11	119.8
C4—C3—C15	120.48 (17)	O1—C12—C13	124.46 (17)
C2—C3—C15	118.32 (17)	O1—C12—C11	115.79 (16)
C5—C4—C3	118.80 (17)	C13—C12—C11	119.74 (17)
C5—C4—H4	120.6	C12—C13—C14	119.34 (17)
C3—C4—H4	120.6	C12—C13—H13	120.3
C6—C5—C4	119.96 (17)	C14—C13—H13	120.3
C6—C5—H5	120.0	C9—C14—C13	121.59 (17)
C4—C5—H5	120.0	C9—C14—H14	119.2
C5—C6—C1	121.04 (17)	C13—C14—H14	119.2
C5—C6—N1	116.61 (16)	N3—C15—C3	177.3 (2)
C1—C6—N1	122.33 (16)	O1—C16—H16A	109.5
C8—C7—N2	111.75 (16)	O1—C16—H16B	109.5
C8—C7—C9	124.52 (17)	H16A—C16—H16B	109.5
N2—C7—C9	123.71 (15)	O1—C16—H16C	109.5
C7—C8—Cl2	124.62 (15)	H16A—C16—H16C	109.5
C7—C8—Cl1	122.71 (15)	H16B—C16—H16C	109.5
Cl2—C8—Cl1	112.66 (11)	N2—N1—C6	111.24 (15)
C14—C9—C10	118.16 (17)	N1—N2—C7	116.40 (15)
C14—C9—C7	120.73 (17)	C12—O1—C16	118.13 (15)
			
C6—C1—C2—C3	0.9 (3)	C14—C9—C10—C11	0.0 (3)
C1—C2—C3—C4	−1.6 (3)	C7—C9—C10—C11	178.04 (17)
C1—C2—C3—C15	178.65 (18)	C9—C10—C11—C12	0.8 (3)
C2—C3—C4—C5	0.3 (3)	C10—C11—C12—O1	178.52 (17)
C15—C3—C4—C5	−179.91 (18)	C10—C11—C12—C13	−1.1 (3)
C3—C4—C5—C6	1.5 (3)	O1—C12—C13—C14	−178.90 (17)
C4—C5—C6—C1	−2.2 (3)	C11—C12—C13—C14	0.7 (3)
C4—C5—C6—N1	179.12 (16)	C10—C9—C14—C13	−0.4 (3)
C2—C1—C6—C5	0.9 (3)	C7—C9—C14—C13	−178.47 (17)
C2—C1—C6—N1	179.54 (17)	C12—C13—C14—C9	0.0 (3)
N2—C7—C8—Cl2	178.27 (14)	C5—C6—N1—N2	−156.50 (17)
C9—C7—C8—Cl2	−3.3 (3)	C1—C6—N1—N2	24.8 (2)
N2—C7—C8—Cl1	−2.2 (2)	C6—N1—N2—C7	−178.37 (15)
C9—C7—C8—Cl1	176.26 (14)	C8—C7—N2—N1	−176.77 (17)
C8—C7—C9—C14	−52.1 (3)	C9—C7—N2—N1	4.8 (3)
N2—C7—C9—C14	126.2 (2)	C13—C12—O1—C16	−4.7 (3)
C8—C7—C9—C10	129.9 (2)	C11—C12—O1—C16	175.67 (16)
N2—C7—C9—C10	−51.9 (3)		

### Hydrogen-bond geometry (Å, º)

**Table:**

*D*—H···*A*	*D*—H	H···*A*	*D*···*A*	*D*—H···*A*
C2—H2···O1^i^	0.95	2.47	3.391 (2)	165
C16—H16*C*···O1^ii^	0.98	2.59	3.516 (3)	158

Symmetry codes: (i) *x*−1, −*y*+3/2, *z*+1/2; (ii) *x*−1, *y*, *z*.
